# Biomechanical Assessment of Macro-Calcification in Human Carotid Atherosclerosis and Its Impact on Smooth Muscle Cell Phenotype

**DOI:** 10.3390/cells11203279

**Published:** 2022-10-18

**Authors:** Till Seime, Max van Wanrooij, Eva Karlöf, Malin Kronqvist, Staffan Johansson, Ljubica Matic, T. Christian Gasser, Ulf Hedin

**Affiliations:** 1 Vascular Surgery, Department of Molecular Medicine and Surgery, Karolinska Institute, 17164 Stockholm, Sweden; 2 Solid Mechanics, School of Engineering Sciences, KTH Royal Institute of Technology, 10044 Stockholm, Sweden; 3 Biochemistry & Cell & Tumor Biology, Department of Medical Biochemistry and Microbiology, Uppsala University, 75123 Uppsala, Sweden

**Keywords:** atherosclerosis, carotid stenosis, calcification, biomechanics, smooth muscle cells

## Abstract

Intimal calcification and vascular stiffening are predominant features of end-stage atherosclerosis. However, their role in atherosclerotic plaque instability and how the extent and spatial distribution of calcification influence plaque biology remain unclear. We recently showed that extensive macro calcification can be a stabilizing feature of late-stage human lesions, associated with a reacquisition of more differentiated properties of plaque smooth muscle cells (SMCs) and extracellular matrix (ECM) remodeling. Here, we hypothesized that biomechanical forces related to macro-calcification within plaques influence SMC phenotype and contribute to plaque stabilization. We generated a finite element modeling (FEM) pipeline to assess plaque tissue stretch based on image analysis of preoperative computed tomography angiography (CTA) of carotid atherosclerotic plaques to visualize calcification and soft tissues (lipids and extracellular matrix) within the lesions. Biomechanical stretch was significantly reduced in tissues in close proximity to macro calcification, while increased levels were observed within distant soft tissues. Applying this data to an in vitro stretch model on primary vascular SMCs revealed upregulation of typical markers for differentiated SMCs and contractility under low stretch conditions but also impeded SMC alignment. In contrast, high stretch conditions in combination with calcifying conditions induced SMC apoptosis. Our findings suggest that the load bearing capacities of macro calcifications influence SMC differentiation and survival and contribute to atherosclerotic plaque stabilization.

## 1. Introduction

Symptomatic carotid artery stenosis caused by unstable atherosclerotic lesions has been estimated to cause 8–20% of all ischemic strokes [1]. Unstable atherosclerotic plaques are characterized by inflammation, a lipid-rich necrotic core (LRNC), intra-plaque hemorrhage, a thin fibrous cap, and depletion of smooth muscle cells (SMCs). Biomechanically, accumulating debris of lipid depositions and cell apoptosis leads to secondary necrosis and necrotic core stiffening [2]. Lesion stiffening is further accentuated by increasing levels of late-stage plaque calcification. In contrast to micro-calcification, as well as early stages of speckled and fragmented vascular calcification that have been related to adverse clinical outcome [3], extensive macro-calcification has rather been reported to be a predominant feature of clinically stable, asymptomatic, lesions [4]. In support of these observations, we have shown that carotid plaques from asymptomatic patients are enriched in biological processes associated with calcification together with classical SMC markers [5]. In addition, we recently demonstrated that macro-calcification can be a stabilizing factor in late-stage human carotid atherosclerosis. Assessment of plaque calcification by computed tomography angiography (CTA) and analyses of plaque gene expression associated macro-calcification with molecular signatures typically linked to plaque stability and in particular genes related to a differentiated SMC phenotype [6,7].

Observing signs of differentiated SMCs in atherosclerosis was an unexpected finding as SMCs within atherosclerotic lesions are generally dedifferentiated with secretory and proliferative capacities, essential for preserving tissue integrity and formation of a protective fibrous cap, while differentiated SMCs normally reside within the tunica media [8,9]. Since differentiated SMCs have been shown to be responsive to biomechanical forces (assessed via typical markers like MYOCD, CNN1 and MYH11) [10], it is possible that macro-calcification in atherosclerotic plaques alters vessel biomechanics and tissue stress with consequences for the phenotypic status of SMCs.

Previously, estimation of structural plaque stresses from computational finite element and in silico modeling (FEM) have indicated that calcification can lower overall tissue stress due to greater stiffness and load bearing capacity [11] and wide-spread macro-calcification might result in advantageous stress redistribution [12]. However, in order to comprehensively assess the distribution of stresses in atherosclerotic lesions, discrimination of plaque tissue components with different biomechanical properties is necessary. Detailed characterization of plaque morphology with determination of LRNC, extracellular matrix-rich tissue (ECM) and macro-calcification can be accomplished by post-processing of computed-tomography angiography (CTA) images utilizing dedicated analytical software [7,13,14,15].

Here, we hypothesized that plaque macro-calcification modulates SMC phenotype due to biomechanical properties and altered tissue stretch. Our hypothesis was evaluated combining in silico FEM of human carotid plaques, derived from clinical CTA image analysis and annotation of different plaque tissue components, with in vitro assays to assess the effect of mechanical stretch and calcification on phenotypic changes in primary human SMCs.

## 2. Materials and Methods

### 2.1. Computed Tomography Angiography Image Analysis

Carotid plaques were assessed in pre-operative CTA from patients (*n* = 4) with symptomatic and asymptomatic carotid artery stenosis using a semi-automated, histology-validated software as previously described (ElucidVivo^®^ version A.2 Oct 8 2019, Elucid Bioimaging Inc., Boston, MA, USA) [13,14,15,16,17,18]. Four patients were randomly selected from a cohort of patients that underwent stroke-preventive carotid endarterectomy at Karolinska University Hospital between 2008–2012 (Table 1). The cohort included patients with a high ratio of calcification of the carotid plaque as assessed by pre-operative CTA [6] (Table 1). In brief, reconstructed images were analyzed in a blinded fashion by one observer (E.K.) to characterize plaque structure and composition (plaque morphology) [7] creating 3D segmentations with improved resolution and soft tissue plaque component differentiation. A patient-specific 3D point spread function restored image intensities to represent the original tissues, which mitigates artefacts and enables discrimination of tissue types such as LRNC, calcification (CALC) and tissue types representing plaque tissue not detected as either LRNC or CALC (MATX). To avoid limitations of fixed thresholds, accuracy was achieved by algorithms that account for distributions of tissue constituents rather than assuming constant material density ranges. The common and internal carotid artery were defined as target, lumen and wall evaluated automatically or edited manually when needed.

### 2.2. In Silico Biomechanical Modeling

FEM analysis of the four patients was conducted using COMSOL Multiphysics 5.6 (COMSOL Inc., Burlington, MA, USA),. In brief, 3D segmented images from ElucidVivo® (version A.2 Oct 8 2019, Elucid Bioimaging Inc., Boston, MA, USA) were converted to the common STL format using the open source software iso2mesh (version 1.9, by Qianqian Fang) [19]. Slight surface smoothing was applied to avoid local mesh irregularities, the generated STL surface mesh was imported into COMSOL and then converted into a 3D solid domain of the vessel wall. The domain was then represented by approximately 20 k quadratic tetrahedral finite elements. A spatial interpolation function was used to specify the local tissue characteristics, i.e., LRNC, CALC and MATX. The mechanics of each of these tissues were modeled by a three-parameter Yeoh strain energy density function applying material parameters based on previous research (Table 2); for a full summary of plaque tissue properties, please refer to [20]. The Lagrange constraint method was used to enforce tissue incompressibility. Diastolic (set at 80 mmHg) and respective systolic (set at 140 mmHg) blood pressures were applied to the wall and the displacement field calculated by solving the quasistatic equilibrium equations. Tissue stretch was then assessed at three cross-sections distal to the bifurcation, covering areas before, within and after maximal stenosis.

### 2.3. In Vitro Cyclic Stretch Assay

Commercial primary human aortic smooth muscle cells (HAoSMCs) from one male donor with no reported cardiovascular comorbidities were obtained from Lonza (#CC-2571, Lot No. 0000369150, ascending aorta, Basel, Switzerland) and subjected to 0% or 12.5% substrate stretch as previously described [21]. In brief, custom made silicon chambers (inner dimensions: 4 cm^2^ or 3 cm^2^) with replaceable silicon membrane bottoms were sterilized and coated with human fibronectin (5 μg/cm^2^, #PHE0023, Life Technologies, Carlsbad, CA, USA). HAoSMCs at passage 6 were plated at sub-confluence (50,000 cells per cm^2^) and left to adhere overnight in basal growth medium (5% FBS, #CC-3181, Lonza, Basel, Switzerland). After 4 h of serum starvation in OptiMEM medium containing 0.1% FBS (#51985-026, Life Technologies, Carlsbad, CA, USA), the medium was changed to OptiMEM medium containing 1% FBS and 1.5 mM Ca, 1.5 mM PO_4_. Controls were treated identically and supplement volumes replaced with PBS. Axial cyclic stretch was applied to the silicone chambers with a frequency of 70 cycles per minute. For comparison, HAoSMCs were also plated in fibronectin coated regular 6-well plates for expression analysis and chamber slides for immunofluorescence (IF) imaging, under identical medium conditions. After 8, 24 and 48 h medium supernatant, cell lysates or complete membranes were collected for further analysis.

### 2.4. RNA Extraction and Gene Expression Analyses by Quantitative PCR (qPCR)

RNA was prepared using RLT (#79216, Qiagen, Venlo, The Netherlands) buffer containing 1% 2-Mercaptoethanol (M3148, Sigma-Aldrich, St. Louis, MO, USA) and purified by the RNeasy Mini kit (#74106, Qiagen, Venlo, The Netherlands), including DNase digestion. For qPCR, total RNA was reverse-transcribed using High-Capacity RNA-to-cDNA kit (#4387406, Applied Biosystems, Carlsbad, CA, USA). PCR amplification was performed in 384-well plates in a 7900 HT real-time PCR system (Applied Biosystems, Carlsbad, CA, USA), using TaqMan^®^ Universal PCR Master Mix (#4324018, Applied Biosystems, Carlsbad, CA, USA) and TaqMan^®^ Gene Expression Assays (MYOCD probe Hs00538076_m1; ACTA2 probe Hs00426835_g1; MYH11 probe Hs00975796_m1; CNN1 probe Hs00959434_m1; LMOD1 probe Hs00201704_m1; PDLIM7 probe Hs00193775_m1; HSPG2 probe Hs01078536 m1; TAGLN probe Hs01038773_g1; Thermo Fisher, Waltham, MA, USA). All samples were measured in duplicate. Results were normalized to the equal mass of total RNA as well as the Ct values of RPLPO (Hs99999902_m1; Thermo Fisher, Waltham, MA, USA) housekeeping control. The relative amount of target gene mRNA was calculated by the 2^−ΔΔCt^ method.

### 2.5. Immunofluorescence

Primary cells were fixed in 4% Zink-formaldehyde and directly processed on the silicone membranes or chamber slides, respectively. Non-specific binding was blocked with 2% BSA in 0.1% Triton X-100/PBS. Samples were then incubated with primary antibodies (SMA, A-5691, Sigma-Aldrich, St. Louis, MO, USA; LMOD1, HPA030097, Sigma-Aldrich, St. Louis, MO, USA; MYH11, ab133567, Abcam, Cambridge, UK; cleaved CASP3, 9661S, Cell Signaling Technology Inc., Danvers, MA, USA) diluted in blocking buffer, washed with PBS and counterstained with Alexa Fluor 568-conjugated secondary antibodies (Invitrogen, Carlsbad, CA, USA). Isotype-matched rabbit and mouse IgG were used as negative controls. Nuclei were stained with diamidino-2-phenylindole (DAPI). Membranes were mounted on slides cell-side down with fluorescent mounting medium (#S3023, DAKO, Santa Clara, CA, USA). Images were taken using a Leica TCS SP8 confocal laser scanning microscope (Leica Microsystems GmbH, Wetzlar, Germany) and Nikon Eclipse E800 (Nikon Instruments Inc., Melville, NY, USA) microscope equipped with a pE-300 lite light source (CoolLED ltd., Andover, UK).

### 2.6. Statistical Analyses

Comparative statistics between time-points and groups were conducted using 2-way ANOVA or simple comparison between groups by 1-way ANOVA. All statistical analyses were performed with GraphPad Prism 9.

## 3. Results

### 3.1. In Silico Modeling of Biomechanical Stretch Distributions among the Major Plaque Components

**Figure 1:**
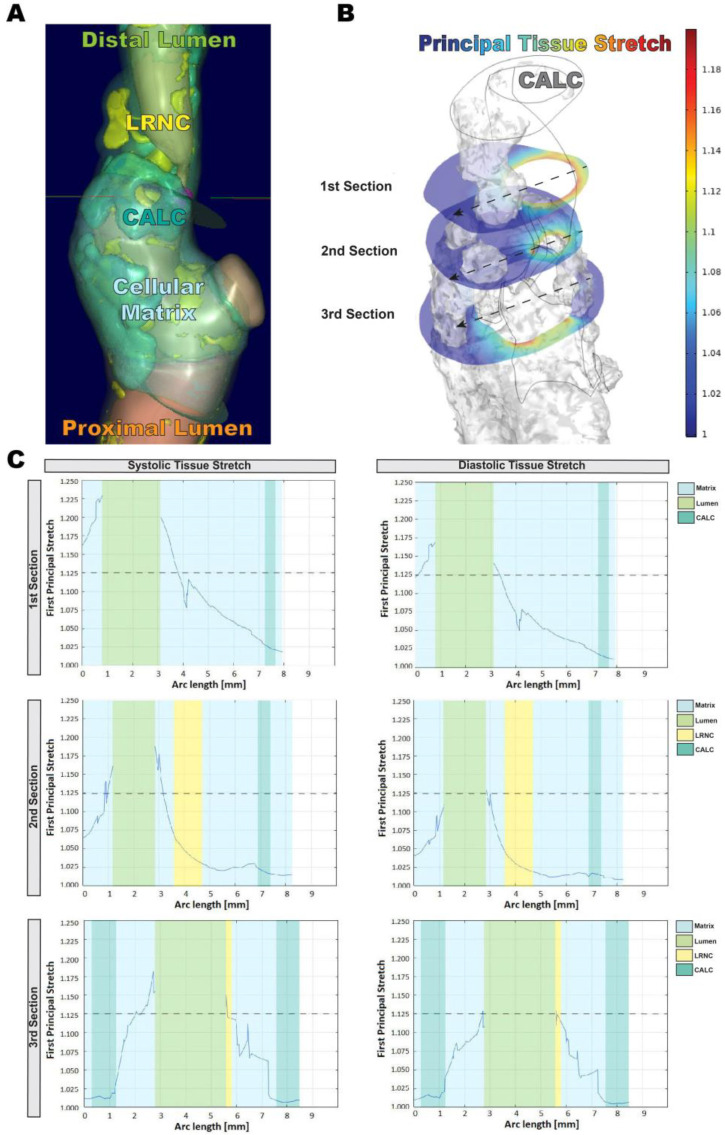
In silico modeling workflow and identification of load bearing capacities by carotid plaque macro-calcification. (**A**) Tissue composition of human carotid plaques was rendered and annotated using the ElucidVivo® (version A.2 8 October 2019, Elucid Bioimaging Inc., Boston, MA, USA) CTA image analysis software. (**B**) FEM analysis was conducted using COMSOL Multiphysics 5.6 (COMSOL Inc., Burlington, MA, USA) to calculate spatial tissue stretch within general plaque components. Illustrations show identical plaque areas for side-by-side comparison. (**C**) Principal tissue stretch was assessed on three cross-sections distal of the bifurcation in relation to the respective tissue properties of CALC, LRNC and matrix. Graphs show distribution of tissue properties and corresponding principal stretch along a center-line, crossing the vessel wall and lumen at the respective cross-section. The dotted line indicates 12.5% principal stretch. Data presented in this figure are derived from one patient and used as a representative dataset of the whole analysis *n* = 4. CALC—calcification (turquoise), LRNC—lipid rich necrotic core (yellow), Matrix—Cellular Matrix (light blue), Distal Lumen (light green), Proximal Lumen (orange).

In silico modeling of four highly calcified carotid lesions was based on quantitative morphological tissue composition, assessed by image analysis of pre-operative CTA (Figure 1A), utilizing the histologically validated ElucidVivo^®^ software (version A.2 Oct 8 2019, Elucid Bioimaging Inc., Boston, MA, USA). The principal tissue stretch under systole and diastole was calculated by FEM analysis in relation to the locations of either CALC or soft tissues (LRNC or MATX; Figure 1B). Based on this model, the principal tissue stretch was markedly reduced within and in close proximity to macro-calcifications compared to soft tissue components, as analyzed on three cross-sections distal to the bifurcation (Figure 1C).

### 3.2. Changed Tissue Biomechanics Alter SMC Alignment and Phenotype In Vitro

To study the impact of plaque biomechanics on SMC phenotype, we applied the calculated tissue stretch values in an in vitro model.

We determined 12.5% substrate stretch to be representative of soft tissues distant (>1 mm) of CALC, while the matrix in close proximity (<1 mm) to CALC was not subject to significant stretch. Therefore, soft tissue biomechanics were modeled in vitro by either 12.5% uniaxial cyclic stretching of flexible silicone membranes with a frequency of 70 iterations per minute or unstretched silicone membranes. For comparison, we also included a condition resembling cells in direct contact with the very stiff calcified surface, a rigid cell culture plate.

**Figure 2:**
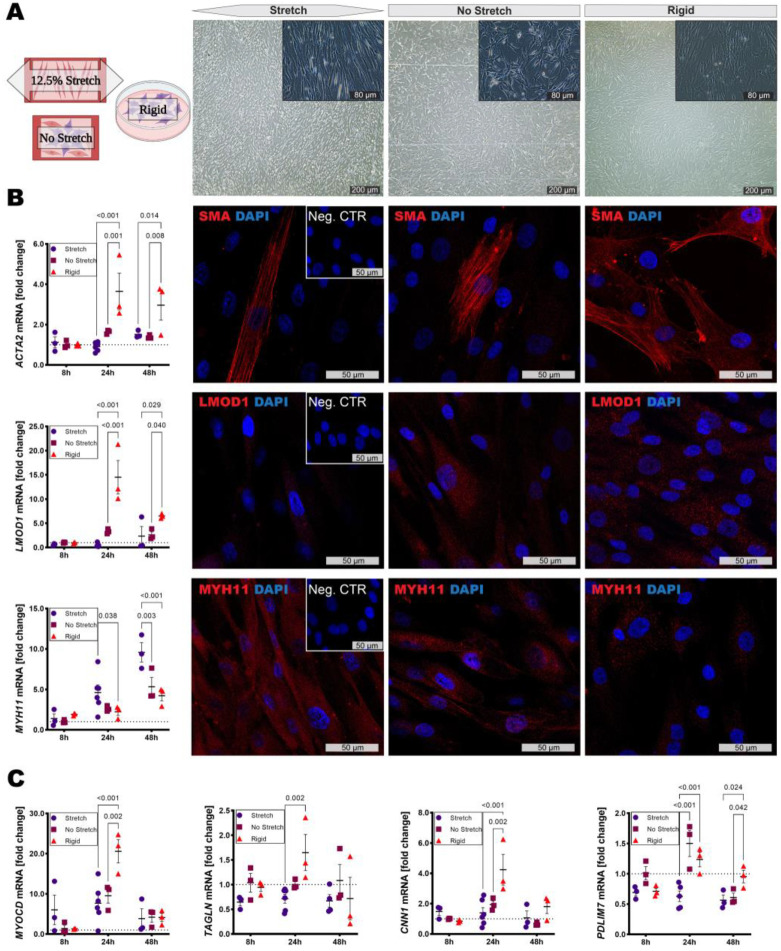
High stretch induces SMC alignment but downregulates most markers of differentiated SMCs. (**A**) Representative images of vascular SMCs after 8 h on stretched/unstretched silicone chambers or rigid culture plates. (**B**) ACTA2/smooth muscle actin as well as LMOD1 were upregulated in cells grown on tissue culture plates as compared to silicon membranes. However, MYH11 showed the opposite trend. Representative images show 24 h. (**C**) *MYOCD*, *TAGLN* and *CNN1* transcript levels were elevated after 24 h in SMCs grown on rigid plates, while *PDLIM7* mRNA expression was most highly upregulated after 24 h under non-stretch conditions. Images show (**A**) 4×/20× magnification and (**B**) 63×. Insets show corresponding isotype negative control. Plots show mean with SEM. Statistical difference assessed by two-way ANOVA. Expression levels calculated according to ΔΔCT and normalized to unstretched conditions at 8 h.

SMCs subjected to biomechanical stimulation showed alignment perpendicular to the direction of stretch already after 8 h and maintained this morphology after 24 h and 48 h. No such alignment could be observed for SMCs grown without stretch or on rigid culture plates (Figure 2A). *ACTA2* as well as *LMOD1*, previously connected to smooth muscle contraction, showed a trend to be upregulated after 24 h without stretch but only reached significance on rigid plates. However, *MYH11* mRNA expression increased under stretch and was significantly upregulated after 24 h and 48 h. These transcriptomic findings were confirmed by representative immunofluorescence stains (IF) (Figure 2B). The general SMC lineage marker *MYOCD* as well as *TAGLN* and *CNN1* mRNA levels were increased after 24 h on rigid plates compared to silicone membranes, while *PDLIM7*, centrally involved in actin cytoskeleton formation, was also upregulated in SMCs growing under unstretched conditions (Figure 2C). Additionally, *HSPG2* expression, encoding the basement membrane proteoglycan perlecan, was significantly increased after 8 h and 24 h in cells on unstretched membranes.

### 3.3. High Stretch Combined with Calcifying Conditions Induces SMC Apoptosis

**Figure 3:**
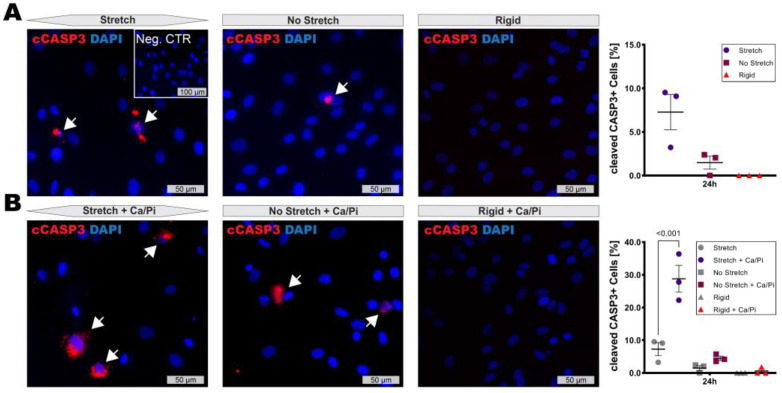
Combination of increased substrate stretch with calcifying conditions induces SMC apoptosis. (**A**) SMC apoptosis marked by cleaved CASP3 signal (white arrows) was marginally induced under stretch after 24 h. (**B**) Combination of stretch and calcifying conditions (1.5 mM Ca and 1.5 mM Pi) strongly induced apoptosis while non-stretched conditions remained unaffected. Images show 40× magnification. Inset shows corresponding isotype negative control. Plots show mean with SEM. Statistical difference assessed by one-way ANOVA.

High stretch conditions caused by hypertension are known to induce SMC dedifferentiation and apoptosis. Accordingly, a slight increase in cleaved CASP3 activity after 24 h of stretch was detectable by IF staining, while almost no signal was present at 8 h or within unstretched controls (Figure 3A). Nevertheless, little is known about the effect of extensive stretch on SMCs under calcifying conditions. Interestingly, SMCs subjected to 12.5% stretch in combination with high calcium and phosphate conditions showed a strong induction of apoptosis after 24 h (Figure 3B) and proceeded to detach, making further analysis at 48 h impossible. The SMC apoptosis rate remained unaffected under low stretch conditions.

## 4. Discussion

In this study, we identified a significant impact of changed tissue biomechanics on SMC phenotype, as a result of extensive macro-calcification within late-stage atherosclerotic lesions. Utilizing a unique pipeline to generate tissue specific FEM analysis from preoperative CTA images and testing our findings in an in vitro model on primary human SMCs, we showed that: (i) Macro-calcification exhibited load-bearing capacities within advanced plaques, generating zones of reduced tissue stretch in close proximity to calcifications, while extracellular matrix- and lipid-rich soft tissues distant from macro-calcified areas were exposed to increased stretch. (ii) SMCs subjected to low stretch conditions, similar to the macro-calcified environment, adopted a phenotype characterized by increased expression of cytoskeletal markers typical for differentiated SMCs but also impaired alignment and loss of MYH11. (iii) High stretch conditions, found in plaque tissues distant from macro-calcifications, combined with calcifying conditions led to rapid SMC apoptosis. These findings support our previous discovery of SMC differentiation associated with highly-calcified human carotid plaques [6] and provide a mechanistic link between macro-calcification, tissue biomechanics and SMC phenotype on a patient specific level.

Early micro-calcification, aggregating within the necrotic core, fibrous cap and medial tissue matrix due to local collagen fiber compositions [3], has been identified to create tissue stress concentrations leading to plaque rupture [22]. Nevertheless, our data confirm idealized studies suggesting stabilizing biomechanical properties for extensive macro-calcification [23]. We found that tissue stress was several folds reduced within the soft tissue matrix surrounding highly calcified areas. As a result, SMCs within these zones were subjected to insignificant stretch, whereas they had to sustain an average of 12.5% stretch in other regions of the plaque. Under systole up to a 10% stretch are considered physiological, while this number can increase to 20% in cases of hypertension [24,25]. Pathological conditions exceeding 10% circumferential stretch cause vascular remodeling including induction of SMC apoptosis and fibrotic ECM expression [26,27]. Contractile SMCs express typical cytoskeletal markers such as SMA and MYH11 to actively distribute strain within the arterial wall and maintain contractile tone. They are embedded in a basement membrane, mainly composed of collagen type-IV, laminin and HSPG2, supporting homeostasis [28]. Nevertheless, SMCs retain the capacity of dedifferentiating to a “synthetic” phenotype, characterized by disappearance of the basement membrane, acquisition of migratory and proliferative properties, and expression of structural proteins and matrix degrading enzymes, relevant for ECM remodeling [29]. This feature is crucial to adapt vessel compliance and regain homeostasis after vascular injury. However, sustained pathological conditions will lead to extensive ECM matrix formation and subsequent vessel wall stiffening.

Since neither condition, no stretch vs. an average of 12.5% stretch, can be considered physiological, we investigated their impact on SMC phenotypic changes. In addition to stretchable silicone membranes used to model biomechanics of the soft tissue environment, we included standard rigid culture plates as the closest replication of a calcified plaque matrix which reaches a Young’s modulus of up to 25 GPa [30]. Our experiments confirmed previously published results showing the importance of biomechanical stimulation for SMC alignment [31]. When subjected to 12.5% axial stretch, SMCs aligned perpendicularly to the stretch direction already after 8 h and maintained this general morphology. However, most markers of SMC differentiation and cytoskeletal integrity, including *MYOCD*, *ACTA2*, *TAGLN*, *CNN1*, *LMOD1* and *PDLIM7*, were reduced compared to unstretched conditions. Of note, SMCs growing on rigid culture plates, resembling the biomechanics of a macro-calcified environment, showed the highest expression of these markers. While expression of proteins more generally involved in cytoskeleton formation, such as ACTA2 and CNN1, can be maintained during SMC phenotypic changes, we previously showed that marker genes like *LMOD1* and *PDLIM7* are sensitive indicators of SMC differentiation and a contractile phenotype [32]. Nevertheless, a significant decrease of MYH11 expression in SMCs lacking biomechanical stimulation suggests an incomplete differentiation and contractile function [8,33]. A significant increase in *HSPG2* mRNA expression, encoding for the basement membrane key proteoglycan perlecan [34,35] and closely related to the acquisition of a contractile SMC phenotype [36], in SMCs grown on unstretched membranes suggests that the soft tissue in close proximity to macro-calcifications may be the most favorable biomechanical condition for SMC quiescence and vascular homeostasis in an advanced plaque milieu.

Progressive plaque calcification is classically considered to be a marker for increasing atherosclerotic disease burden [37,38]. Within an atherosclerotic milieu rich in calcium and phosphate, SMCs have been shown to act as key initiators of early calcification via the release of exosomes [39], forming nucleation sites similar to osteoblast-derived matrix vesicles [40]. These microparticles and uncleared debris of apoptotic cells act as the first *nidus* for further mineralization due to their surface-bound Ca binding complexes [41,42]. Our experiments showed that the combination of pathological substrate stretch and calcifying conditions can lead to a severe increase in apoptosis and rapid disintegration of the aligned SMC layer, while SMC survival was maintained without stretch. Since differentiated SMCs have been shown to be particularly susceptible to elevated biomechanical stress [26], it is likely that stretch-induced opening of Ca-ion channels leads to a fatal influx of Ca^2+^ under calcifying conditions, triggering mineral overload, cellular adaptation and apoptosis [43,44].

Collectively, our study highlights a potential pipeline to unravel the connection between plaque composition, biomechanics and molecular processes on a patient specific level. We were able to confirm, based on clinical CTA imaging, that macro-calcifications can support SMC survival and differentiation under atherosclerotic conditions by generating low stretch zones within advanced lesions. This approach could serve to improve patient diagnostics and help to identify and predict atherosclerotic lesion progression via non-invasive imaging modalities.

## 5. Limitations

This study is based on clinical CTA of n = 4 patients and should therefore be seen as proof of concept, not necessarily representative of a population. In order to generate a coherent mesh for FEM, it was in some areas necessary to apply smoothing effects to the ElucidVivo® data due to the relatively low resolution of clinical CTA imaging. Tissue component properties had to be generalized to previously experimentally validated parameters. Additionally, we applied standardized blood pressures and identical tissue biomechanical properties of the individual plaque components across patients. While the chosen values represent common characteristics of medically controlled CVD patients, it should be mentioned that this aspect was not studied on a patient specific level. Furthermore, the complex output matrix of 3D tissue stretch limited the possibilities for statistical evaluation. Primary human aortic and carotid SMCs at low passages were used in this study. While these cells express the typical markers and have functional features of differentiated SMCs, we cannot exclude that some of the more sensitive markers are already downregulated even at the early stage after cell isolation, contributing to the onset of phenotypic modulation.

## Figures and Tables

**Table 1:** Demographics of patients included for CTA and in silico analysis.

Patient Characteristics	Whole Cohort, n
N	4
Age (year, mean)	68
Sex (female/male)	1/3
BMI (mean)	29
Smoking	
Present	1
Former	1
Never	2
Comorbidities	
previous myocardial infarction	1
angina pectoris	2
diabetes	0
Therapy	
lipid lowering (ezetimib, HMG-CoA reductase inhibitors)	4
antidiabetics	0
antihypertensives (ACE inhibitors, beta-blockers, diuretics, angiotensine II blockers)	3
Symptoms	
*Amaurosis fugax*	1
TIA	1
Minor Stroke	0
Asymptomatic	2

**Table 2:** Yeoh material parameters prescribed (in Pa) to describe the individual plaque tissue components.

MATX	CALC	LRNC
c1 = 2.35 10^4^	c1 = 3.02 10^5^	c1 = 2.96 10^4^
c2 = 1.26 10^5^	c2 = −2.28 10^5^	c2 = −3.32 10^4^
c3 = 1.12 10^5^	c3 = 2.61 10^5^	c3 = 1.29 10^5^

## Data Availability

The data that support the findings of this study are available from the corresponding author upon reasonable request.
